# Absorption by the Skin

**Published:** 1875-03

**Authors:** 


Absorption by the Skin.—"A correspondent calls our attention to the fact that while the survivors of the Cospatrick suffered agonies of thirst in their boat, the two men from the La Plata, who were actually in the water up to their waists nearly all the time of their three days' exposure on their damaged raft, felt no thirst and no hunger. Only when the sight of the Dutch vessel—their rescuer—made them feverish with hope and excitement did they begin to find their throats grow parched. Does not a plunge into the sea relieve thirst? our correspondent asks. And he gives his own experience as a swimmer in a seaport town, when he was young, in testimony that it does produce that effect. The water entering the pores relieves the parched-up palate; and, perhaps, as he puts it, a filtration takes place in the process, which gets rid of the presence of the saline matter. The theory is affirmed with great precision, we remember, by one of George Sand's heroes—not a high authority, certainly, on medical or scientific questions; but the experiences of a hero of romance, if only they are adapted from anybody's actual life, are as good, so far, as those of Hippocrates or Huxley. George Sand's hero takes a bath for the purpose of quenching unusual thirst, and avers that it is the best and surest way of allaying pangs which the drinking even of fresh water would only aggravate. The question ought to be very easily settled, and the only wonderful thing about it is, that, if the belief of our correspondent and the French romancist's hero be correct, there should ever have been any question at all. We fear that if so ready and simple a remedy was also sure, it could hardly have failed long ago to find universal recognition. The thirst which is caused by mere heat would be naturally assuaged by a plunge into the water; but the thirst which is born of exposure, exhaustion and want of food is perhaps not so easily conquered or cured. Still there is something in the fact of the singular contrast presented by the two instances our correspondent has noticed, and the one striking difference in the conditions, which perhaps our philosophy might think it worth while to find out."

The above is from the London *Daily News*, under the heading A Hint to Shipwrecked Mariners, and is of interest to the profession as well as to the laity. That the skin is a valuable absorbing agent is unquestionable. (See Flint, *Physiology*, ii., pp. 451–5.) Life has occasionally been sustained in infants by milk baths. The promulgation of information like this among the people is a part of our duty as public health-conservators.

